# Prognostic model of patients with liver cancer based on tumor stem cell content and immune process

**DOI:** 10.18632/aging.103832

**Published:** 2020-08-27

**Authors:** Weikaixin Kong, Miaomiao Gao, Yuchen Jin, Weiran Huang, Zhuo Huang, Zhengwei Xie

**Affiliations:** 1 Department of Molecular and Cellular Pharmacology, School of Pharmaceutical Sciences, Peking University Health Science Center, Beijing, China; 2 Peking University International Cancer Institute and Department of Pharmacology, School of Basic Medical Sciences, Peking University, Beijing, China

**Keywords:** bioinformatics, immunity, liver cancer, prognosis, tumor stem cells

## Abstract

Globally, liver hepatocellular carcinoma (LIHC) has a high mortality and recurrence rate, leading to poor prognosis. The recurrence of LIHC is closely related to two aspects: degree of immune infiltration and content of tumor stem cells. Hence, this study aimed to used RNA-seq and clinical data of LIHC from The Cancer Genome Atlas, Estimation of Stromal and Immune cells in Malignant Tumours, mRNA stemness index score, and weighted gene correlation network analysis methods to find genes significantly linked to the aforementioned two aspects. Key genes and clinical factors were used as input. Lasso regression and multivariate Cox regression were conducted to build an effective prognostic model for patients with liver cancer. Finally, four key genes (*KLHL3*0, *PLN*, *LYVE1*, and *TIMD4*) and four clinical factors (Asian, age, grade, and bilirubin) were included in the prognostic model, namely Immunity and Cancer-stem-cell Related Prognosis (ICRP) score. The ICRP score achieved a great performance in test set. The area under the curve value of the ICRP score in test set for 1, 3, and 5 years was 0.708, 0.723, and 0.765, respectively, which was better than that of other prognostic prediction methods for LIHC. The C-index evaluation method also reached the same conclusion.

## INTRODUCTION

Liver hepatocellular carcinoma (LIHC) is one of the most common malignant tumors with a poor prognosis and has been a global health concern [1]. Worldwide, LIHC is the seventh most common type of cancer and the second most common cause of cancer-related deaths [2]. The World Health Organization has estimated that more than 1 million patients will die from liver cancer in 2030 [3]. The poor prognosis of LIHC is the major factor influencing the mortality of LIHC, with only 18% of 5-year survival [4], which is lower than that of bladder cancer (77.1%), renal pelvis cancer (74.8%), myeloma (52.2%), and so on. Patients often have shorter lifetime and low survival quality after hepatectomy due to the high recurrence rate and metastasis of LIHC [5]. Many factors have been verified to participate in the prognosis of LIHC [6], such as some cell proliferation– and apoptosis-related genes and mTOR pathway–related genes.

Based on the aforementioned studies, a prognostic model, which contained gene expression and clinical factors, was built to predict the prognostic situations of patients with LIHC. The tumor stage, that is American Joint Committee on Cancer (AJCC) stage, was developed to predict the prognosis; however, its practicality still needs to be improved [7]. Further, another novel evaluation system, namely albumin–bilirubin (ALBI) grade [8], was introduced in 2015. This grading system worked well in the measures of liver function or dysfunction, but it included only two factors (bilirubin and albumin). Therefore, a comprehensive prognostic score containing various kinds of information is required, which may have better results than existing approaches.

Accumulating evidence has proved the pivotal role of tumor microenvironment, especially immune-related microenvironment, in tumor progression [9]. A previous study [10] highlighted the importance of immune infiltration in tumor microenvironment for the recurrence and metastasis of LIHC. In addition, evidence showed that CD4:CD8 lymphocyte ratio, high level of infiltrate, and Foxp3+ lymphocytes had a high correlation with LIHC prognosis. Another study [11] demonstrated that tumor-associated macrophages, individually or synergistically with CD45RO^+^ memory cells (TM), could prevent the recurrence and metastasis of LIHC and prolong patient survival. These studies supported high correlations between some important compositions in the microenvironment and LIHC prognosis. The “Estimation of Stromal and Immune cells in Malignant Tumours using Expression data” (ESTIMATE) algorithm introduced by Yoshihara et al was used to infer the fraction of stromal and immune cells in tumor samples [12].

Cancer stem cells (CSCs) are cells within a tumor that possess the ability to self-renew and are responsible for maintaining the growth of the tumor. They contribute in the form of a new tumor colony and produce progeny of multiple phenotypes [13]. Moreover, CSCs express numerous and diverse immune factors, which enable these cells to efficiently modify immune responses to help tumors escape immune-mediated destruction [14]. The biomarkers could be the indication of the development and metastasis of cancer. Previous studies explored the poor prognosis of LIHC caused by CSCs [15]. Malta et al [16] used an innovative one-class logistic regression machine-learning algorithm to provide novel stemness indices for assessing the degree of oncogenic dedifferentiation to evaluate the cancer progression. mRNAsi and EREG-mRNAsi were defined to reflect the gene expression and epigenetic features. This method was employed to evaluate the content of CSCs for samples in the present study.

In this study, both immune infiltration and CSCs were taken into consideration. mRNAsi together with weighted gene co-expression network analysis (WGCNA) was used to score the content of CSCs for the tumor cells from samples collected from The Cancer Genome Atlas (TCGA) database. The ESTIMATE method was introduced to evaluate the degree of immune infiltration. Finally, the ICRP score predicted by the prognosis model was obtained by jointly using multiple information analysis methods. The ICRP score was evaluated in the test set, and its effectiveness was compared with the AJCC stage and ALBI score. The area under the curve (AUC) of the ICRP score in the test set for 1, 3, and 5 years was 0.708, 0.723, and 0.765 respectively, which was obviously higher than that of the AJCC stage and ALBI score. Also, the C-index of the ICRP score in the training and test sets was significantly higher than that of the AJCC stage and ALBI score, indicting the superiority of the proposed model.

## RESULTS

### DEGs related to immune processes

**Figure 1:**
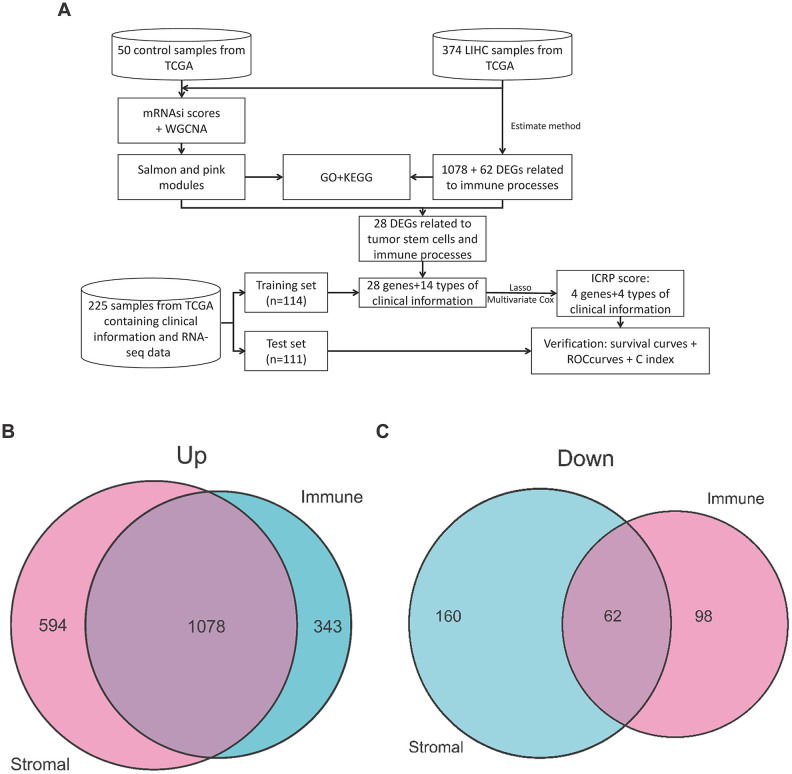
**Workflow of this study and DEGs gene intersection map of the Estimate algorithm in this study.** (**A**) Workflow of this study. (**B**) Intersection map of upregulated genes in StromalScore and ImmuneScore. (**C**) Intersection map of downregulated genes in StromalScore and ImmuneScore.

In order to find important genes closely related to the immune process in LIHC patients, we used StromalScore and ImmuneScore in the Estimate algorithm to evaluate the stromal cell content and immune cell content of the samples. After that, we grouped the samples according to the median of StromalScore and ImmuneScore, screened the DEGs between the high score group and the low score group, and then intersected the DEGs of the two scores. StromalScore's high group has 1672 up-regulated genes (Supplementary Figure 1A), ImmuneScore's high group has 1421 up-regulated genes (Supplementary Figure 1B), and two sets have 1078 intersection genes (Figure 1B). StromalScore's high group has 222 down-regulated genes (Supplementary Figure 1A), ImmuneScore's high group has 160 down-regulated genes (Supplementary Figure 1B), and they have 62 intersection genes (Figure 1C). ImmuneScore has significant differences (Supplementary Figure 1C, *P*=0.029) in different stages, but StromalScore does not show significant difference (Supplementary Figure 1D, *P*=0.067) in different stages.

### Results related to tumor stem cell scoring

**Figure 2:**
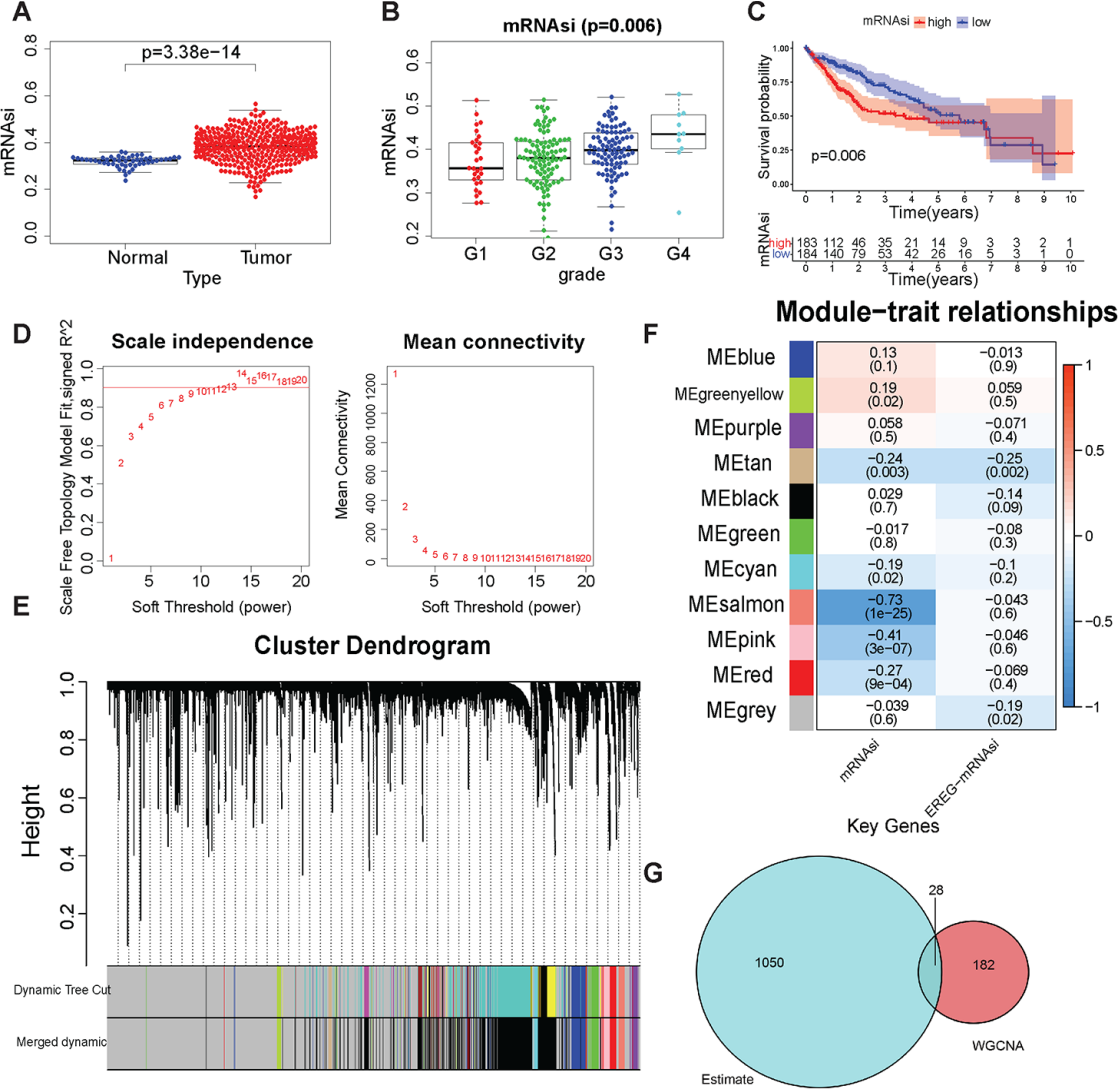
**Tumor stem cell score and WGCNA-related results.** (**A**) mRNAsi in LIHC and normal samples. (**B**) mRNAsi was in different grades. (**C**) Kaplan–Meier plot of high- or low-mRNAsi patients from TCGA. The number of patients remaining at a particular time point is shown at the bottom. (**D**) Left: The determination coefficient *R*^2^ of the *y*-axis is −log_10_ (*k*) and log_10_ (*P*(*k*)). The larger the *R*^2^, the more the gene regulatory network conformed to the scale-free network. *k*, Connectivity of the gene nodes; *P*(*k*), probability of such a node. Red line: 0.9. The *x*-axis is the soft threshold beta. Right: the average connectivity of the *y*-axis genes. The *x*-axis is the soft threshold *β*. (**E**) Gene clustering and gene module partition results. Gene clustering and gene module division results. “Dynamic Tree Cut” is the result before the modules were merged; “Merged dynamic” is the result after the modules were merged. (**F**) The result of the module–trait relationship. The Pearson correlation coefficient of the first principal component of the gene module and the traits was plotted as a heat map, where the *P* values are marked in parentheses. (**G**) The intersection of the ESTIMATE algorithm and mRNAsi + WGCNA-derived genes. These 28 genes were considered to be closely related to the tumor stem cell content and the degree of immunity.

The content of tumor stem cells in tumor tissues had a strong correlation with tumor recurrence, often leading to poor prognosis. The prediction results of tumor stem cell content (mRNAsi) of TCGA samples by Malta et al were used to evaluate the content of tumor stem cells in samples [16]. The mRNAsi was analyzed in different clinical traits. A significant difference (Wilcoxon test, *P* < 0.001) in mRNAsi was found between LIHC samples and normal samples (Figure 2A). mRNAsi did not differ significantly in different ages, genders, and tumor stages (Supplementary Figure 2A–2C). In different tumor grades, mRNAsi has significant differences (Figure 2B, Wilcoxon test, *P=*0.006), and with the increase of grade, the mRNAsi score gradually increases, which shows that mRNAsi can fully reflect the tumor cell differentiation status. The data of patients with LIHC obtained from TCGA were grouped according to the median mRNAsi value, and the Kaplan–Meier test was performed for survival analysis to explore the relationship between mRNAsi and patient prognosis. Patients with low mRNAsi scores had a better prognosis (Figure 2C, *P*=0.006). The results showed that it was reasonable to use mRNAsi to evaluate the tumor stem cell content in LIHC samples and normal samples.

DEGs in 50 normal tissues and 374 LIHC tissues were first screened to find genes in LIHC samples that were closely related to disease occurrence and tumor stem cell scoring. A total of 7273 upregulated genes and 394 downregulated genes were obtained in tumor tissues (Supplementary Figure 2D and 2E). Then, these DEGs were used for WGCNA and mRNAsi as the phenotype for analysis. In this study, when the soft threshold *β* = 13, the gene regulatory network better met the scale-free network (Figure 2D). First, 7667 DEGs were used to perform hierarchical clustering on 374 LIHC samples. Samples with “Height” greater than 1500 were regarded as outliers and excluded (Supplementary Figure 3A, 3B). Then, the genes were clustered and the gene modules were merged. The “mergeCutHeight” value was set to 0.4 to merge the different gene modules obtained (Supplementary Figure 4A). Figure 2E shows the results of gene clustering. “Dynamic Tree Cut” shows the gene modules before merging, and “Merged dynamic” shows the gene modules after merging. The correlation between the mRNAsi and the first principal component of the gene modules (Pearson's correlation coefficient) is shown in Figure 2F. The pink module (–0.41, *P* < 0.001) and the salmon module (–0.73, *P* < 0.001) were closely related to mRNAsi. Moreover, both correlation coefficients were negative, indicating that a high expression of genes in these modules meant lower mRNAsi scores. The relationship between gene expression, phenotype, and the first principal component of the pink and salmon modules are shown in Supplementary Figure 4B and 4C.

### GO and KEGG enrichment analysis

The GO and KEGG enrichment analysis were applied to further evaluate the functions and mechanism of these DEGs. The DEGs obtained from the ESTIMATE algorithm were obviously associated with immune-related biological processes, including leukocyte migration and regulating cell surface receptor signaling pathway in immune response; Cellular component analysis showed that these DEGs significantly enriched in extracellular matrix, side of membrane and so on; Molecular function also indicated these genes involved in immune process, such as antigen binding, glycosaminoglycan binding and cytokine receptor activity (Supplementary Figure 5A). Meanwhile, KEGG analysis suggested DEGs were enriched in immune related pathway, like cytokine-cytokine receptor interaction, chemokine signaling, etc. (Supplementary Figure 5B).

**Figure 3:**
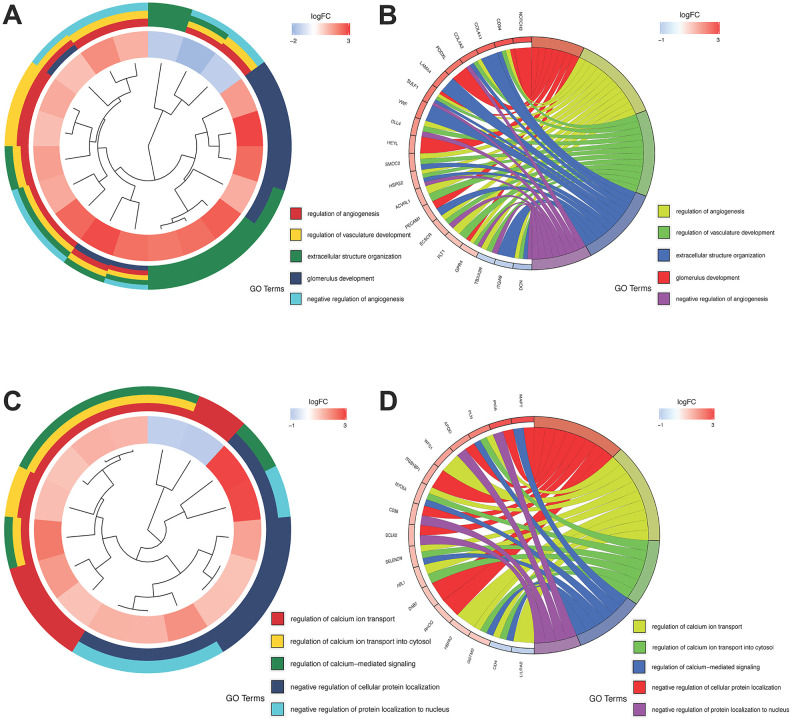
**GO analysis of the salmon and pink modules.** (**A** and **B**) GO analysis of the salmon module; (**C** and **D**) GO analysis of the pink module.

WGCNA indicated that the salmon and pink modules were the significant gene modules related to tumor development. The GO analysis showed that genes in the salmon module were associated with glomerulus development, regulation of angiogenesis and vasculature development (Figure 3A, 3B) while genes in the pink module were predominantly involved in cellular protein localization and calcium ion transport in biological process (Figure 3C, 3D). Remarkably, angiogenesis related to cancer development and calcium homeostasis had an impact on cancer proliferation and metastasis [28].

### Establishment of the prognostic model

The coefficients of the pink and salmon modules obtained by WGCNA were negative (Figure 2F), indicating that the high expression of genes in these two modules represented a decrease in tumor stem cells. The high expression of upregulated genes obtained by the ESTIMATE algorithm meant an increase in the content of immune cells. The reduction in stem cells and the increase in immune cell content were often closely related. Therefore, for finding genes closely related to tumor stem cell content and tumor immune processes, 210 genes in the salmon and pink modules were intersected with 1078 upregulated genes in the ESTIMATE algorithm and 28 key genes were obtained. These 28 genes were used in the construction of subsequent prognostic models. Besides these 28 genes, 14 types of clinical information were also included in the prognostic model to make the prognostic model more informative. A total of 225 patients were then grouped into a training set (*n* = 114) and a test set (*n* = 111). The clinical baseline data and grouping of patients are shown in Table 1. The differences in clinical variables between the training set and the test set were examined to illustrate the rationality of random grouping. The chi-square test was used for discrete variables, while the Wilcoxon test was used for continuous variables. Albumin showed a significant difference between the training and test sets (Table 1, *P* < 0.05), while the other variables showed no significant difference. It was reasonable to have few different variables when grouping because a variety of clinical information was included.

**Table 1:** Clinical baseline data for patients with LIHC.

**Characteristic**	**Train sets (n=114)**	**Test sets (n=111)**	**t/χ2 value**	**P value**
Age	59.07±12.45	59.28±12.33	-0.1266	0.8994
Gender (%)			0.0784	0.3098
Female	40 (35.1)	36 (32.4)		
Male	74 (64.9)	75 (67.6)		
Grade (%)			3.2991	0.3098
G1	14 (12.3)	7 (6.3)		
G2	47 (41.2)	55 (49.5)		
G3	46 (40.4)	44 (39.6)		
G4	7 (6.1)	5 (4.5)		
Stage (%)			6.8583	0.3098
I	74 (64.9)	56 (50.5)		
II	27 (23.7)	29 (26.1)		
III+IV	13 (11.4)	26 (23.4)		
Race (%)			2.3435	0.3098
ASIAN	59 (51.8)	55 (49.5)		
BLACK	6 (5.3)	2 (1.8)		
WHITE	49 (43.0)	54 (48.6)		
Height	165.85±9.06	166.29±13.06	-0.2925	0.7702
Weight	70.82±17.39	74.93±22.36	-1.5425	0.1244
BMI	25.64±5.47	27.6±12.06	-1.5778	0.116
Album	3.67±1.01	3.98±1.05	-2.2608	0.0247
Bilirubin	0.75±0.41	0.92±0.97	-1.6711	0.0961
Creatinine	1.0±0.67	1.6±5.1	-1.2366	0.2176
Fetoprotein	8590.99±37146.93	22260.09±193511.36	-0.7404	0.459

**Figure 4:**
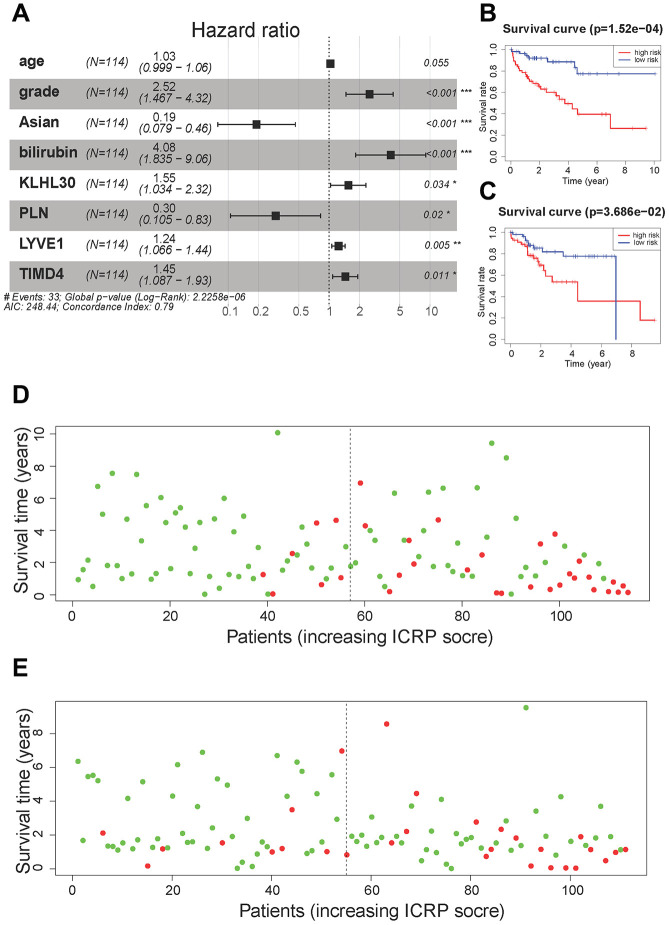
**Cox regression model results.** (**A**) A forest plot of the multivariate Cox regression model. Hazard ratio is provided in the figure. (**B**) The survival curve of the ICRP score in the training set. Grouping was based on the median ICRP score in the training set. Red is the high-level group, and blue is the low-level group. (**C**) The survival curve of the ICRP score in the test set. Grouping was based on the median ICRP score in the training set. (**D**) Patient survival status in the training set. The *x*-axis is the patient ranking in ascending order by the ICRP score; the *y*-axis is the survival time. The red dots are the dead patients, and the green dots are the surviving patients. (**E**) Patient survival status in the test set.

For 28 + 14 variables, lasso regression was first performed to eliminate collinearity between the variables. When the penalty coefficient λ = 0.064, the equation had the smallest error (Supplementary Figure 6A), and the coefficients of nine variables were not 0 (Supplementary Figure 6B). Nine variables were used to build a multivariate Cox regression model, and the independent variable selection method was the back-off method. Finally, eight variables were included in the Cox regression model (Figure 4A). model contains clinical information and genes related to the immune process and CSC, we call this model Immunity and CSC related prognosis (ICRP) score. The formula of the ICRP score was as follows:

$$\begin{array}{l} \text{ICRP score}=0.0306\times \text{Age}+0.9233\times \text{Grade}\\ \;\;\;-1.6563\times \text{Asian}+1.4053\\ \;\;\;\;\;\;\;\;\;\;\;\;\;\;\;\;\;\;\;\;\;\;\;\;\;\;\;\;\;\times \text{Bilirubin}+0.4386\times \text{KLHL}30\\ \;\;\;\;\;\;\;\;\;\;\;\;\;\;\;\;\;\;\;\;\;\;\;\;\;\;\;\;-1.2207\times \text{PLN}+0.2151\\ \;\;\;\;\;\;\;\;\;\;\;\;\;\;\;\;\;\;\;\;\;\;\;\;\;\;\;\;\;\times \text{LYVE}1+0.3701\times \text{TIMD}4 \end{array}$$

Where Age is in year; Grade is an integer from 1 to 4; Asian takes values 1 (means “yes”) and 0 (means “no”); and Bilirubin is in mg/dL; the levels of the four genes are the normalized values of FPKM.

The training and test sets were grouped according to the median value (1.08) of the ICRP score in the training set. All patients with an ICRP score less than 1.08 were in the low-risk group, while all patients with an ICRP score greater than 1.08 were in the high-risk group. Moreover, Kaplan–Meier curves were plotted for survival analysis. The high ICRP score had less survival time in both the training set (Figure 4B, *P* < 0.001) and the test set (Figure 4C, *P* < 0.05). In the training set, the 1- and 3-year OS rate was 94.2% (95% CI = 88.0–100.0) and 85.5% (95% CI = 70.7–98.1) in the low-risk group and 76.6% (95% CI = 66.2–88.6) and 56.9% (95% CI = 44.0–73.6) in the high-risk group, respectively. In the test set, the 1- and 3-year OS rates were 92.2% (95% CI = 85.1–99.8) and 81.8% (95% CI = 70.8–94.6) in the low-risk group and 84.8% (95% CI = 75.6–95.1) and 48.9% (95% CI = 31.3–66.5) in the high-risk group, respectively.

The patients were sorted according to the ICRP score, and the distribution of patient survival status was plotted. In the training set (Figure 4D) and test set (Figure 4E), the survival time of patients gradually decreased and the number of death cases gradually increased with the increase in the ICRP score.

### Verification of the ICRP score

**Figure 5:**
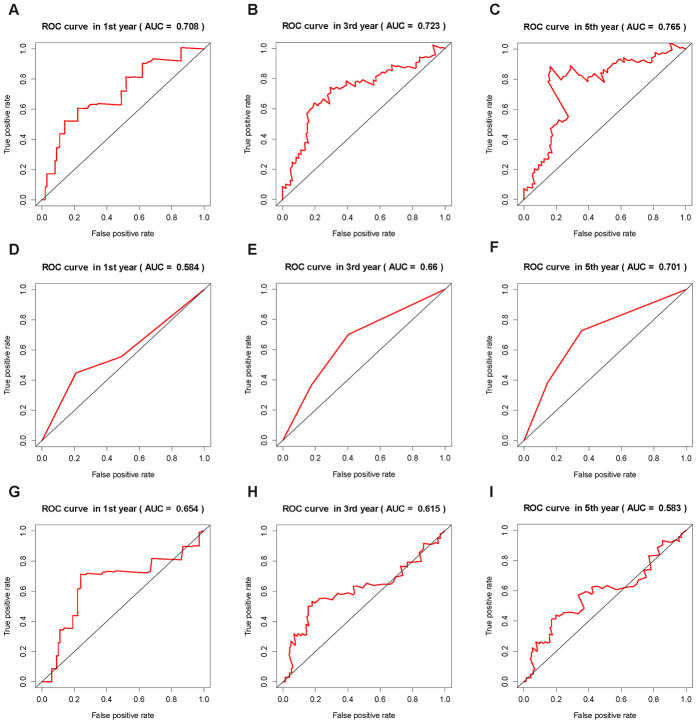
**ROC curves of the ICRP score, AJCC stage, and ALBI score.** The AUC value is in brackets. (**A**–**C**) ROC curves of ICRP score’s forecast result after 1, 3, and 5 years in the test set. (**D**–**F**) ROC curves of AJCC stage forecast result after 1, 3, and 5 years in the test set. (**G**–**I**) ROC curves of ALBI score prediction results after 1, 3, and 5 years in the test set.

In order to further verify the effectiveness of our prognostic model and compare it with other method, we used ICRP score, AJCC stage and ALBI score in the training set and test set to predict the survival status of patients at 1, 3, and 5 years, respectively, and plotted the receiver operator characteristic curves. The area under the curve (AUC) of ICRP score in the test set for 1, 3, and 5 years was 0.708, 0.723, and 0.765 respectively (Figure 5A–5C); the AUC of AJCC stage in the test set for 1, 3, and 5 years was 0.584, 0.660, and 0.701, respectively (Figure 5D–5F); the AUC of ALBI score in the test set for 1, 3, and 5 years was 0.654, 0.615, and 0.583, respectively (Figure 5G–5I). The AUCs of ICRP score in the training set for 1, 3, and 5 years was 0.840, 0.801, and 0.824 respectively (Supplementary Figure 6C–6E); the AUCs of AJCC stage in the training set for 1, 3, and 5 years was 0.592, 0.599, and 0.582 (Supplementary Figure 6F–6H); the AUCs of ALBI score in the training set for 1, 3, and 5 years was 0.641, 0.549, and 0.529 (Supplementary Figure 6I–6K). It can be seen that ICRP score’s prediction ability was better than AJCC stage and ALBI score. To further demonstrate this, the C-index of the ICRP score, AJCC stage, and ALBI score in the training and test sets were calculated. The related results are shown in Table 2. It was obvious that the C-index of the ICRP score was significantly larger than the C-index of the AJCC stage and ALBI score in the training set (*P* < 0.05) and the test set (*P* < 0.05). Carter et al [27] used the TCGA data to establish a signature that could measure chromosomal instability in tumor cells based on the expression of 25 genes, namely CIN25. They proved that CIN25 was an effective prognostic indicator for many cancers [29, 30]. The CIN25 score of each sample was calculated and the patient's prognosis was predicted. The C-index of CIN25 was significantly lower than that of the ICRP score in the training set (*P* < 0.05, Table 2) and test set (*P* < 0.05, Table 2). The aforementioned results showed that the proposed prognostic model was effective.

**Table 2:** C-index results of the ICRP score, AJCC stage, ALBI score, and CIN25.

**Method**	**Training set**		**Test set**	
	C-index(95%CI)	P	C-index(95%CI)	P
ICRP score	0.793(0.713,0.872)		0.697(0.587,0.807)	
AJCC stage	0.561(0.467,0.655)	<0.05	0.570(0.455,0.687)	<0.05
ALBI score	0.602(0.510,0.694)	<0.05	0.605(0.536,0.674)	<0.05
CIN25	0.643(0.579,0.707)	<0.05	0.627(0.564,0.690)	<0.05

In addition, the genes selected in the ICRP score might have false-positive results. Therefore, a sensitivity test was conducted on the genes in the ICRP score. We used 28 randomly selected genes from DEGs in LIHC patients and 14 kinds of clinical information to construct a new prognostic model applying the same process and parameters, and repeated three times. The C-indexes of the three reconstructed prognostic models were calculated and compared with that of the ICRP score. The results of the ICRP score for the training and test sets were significantly greater than those of the three models (*P* < 0.05, Table 3), indicating that the genes selected using the mRNAsi score, WGCNA, and ESTIMATE methods were credible.

**Table 3:** Sensitivity test results of genes in the ICRP score.

**model ID**	**term**	**coef**	**HR**	**HR.95L**	**HR.95H**	**pvalue**	**C index in training set**	***P***	**C index in test set**	***P***	**Randomly selected genes**
model 1	Asian	-1.790	0.167	0.063	0.443	0.000	0.699±0.054	<0.05	0.556±0.050	<0.05	P2RY10 TRBV4-1 PLXNA4 HOXB3 C1QA PCDH7 PLA2G4A PLCB2 CNTN4 RHBG KLK4 PTPRB COL12A1 HAPLN3 GLUL TRDV1 AC011479.1 FAM83E KRT19 COL4A2 ANOS1 BX649601.1 IGHJ3 CSPG4 PTGIR ELK3 MYCT1 ADAMTS12
	fetoprotein	-0.557	0.573	0.300	3.000	0.095					
	CNTN4	-2.772	0.063	0.002	2.121	0.093					
model 2	grade	1.215	3.370	1.819	6.245	0.000	0.712±0.038	<0.05	0.569±0.053	<0.05	TGFA CD300A MFGE8 SLFN12L PDZRN3 FEZ1 GLUL TRIM22 ID3 SELL MMRN2 GPRIN3 NCF1B CXCL14 KCNN3 PSCA C1QTNF3 TRBV4-1 VIM RAB25 POU2F2 DNAJB3 FCRLA IGHV1-69 ADCY3 HOXB3 ARHGAP25 NCF2
	stage	0.576	1.780	1.115	2.841	0.016					
	Asian	-2.272	0.103	0.037	0.289	0.000					
	height	-0.058	0.944	0.909	0.980	0.003					
	TRBV4-1	0.612	1.844	1.112	3.058	0.018					
	HOXB3	-0.667	0.513	0.211	1.248	0.084					
	GLUL	-0.001	0.999	0.998	1.000	0.083					
model 3	age	0.057	1.058	1.018	1.100	0.004	0.678±0.066	<0.05	0.597±0.060	<0.05	C1QTNF7 CST7 CDX1 TRAV12-3 AC090409.1 TRBV7-6 TGFB2 HLA-DQA2 BHLHE22 AC119044.1 DGKA AEBP1 PEG10 PTGIS ST8SIA4 GALNT5 ICAM3 SRGN HBD CSF1R RAB31 LRRC4 ZNF469 TUBB6 ZBP1 MYOM2 STEAP4 AC002398.2
	grade	1.330	3.780	1.759	8.124	0.001					
	Asian	-1.028	0.358	0.113	1.133	0.080					
	BMI	0.045	1.046	1.005	1.088	0.026					
	CDX1	-1.237	0.290	0.085	0.657	0.094					
	CSF1R	0.037	1.038	0.998	1.080	0.063					

**Figure 6:**
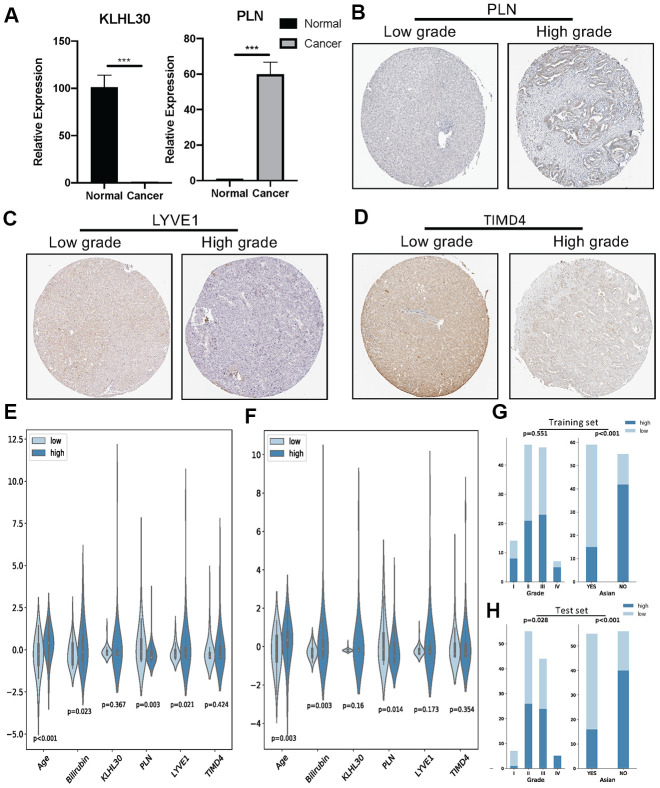
****(**A**) QPCR analysis of KLHL30 and PLN in normal liver cell line and LIHC cell line (*n* = 3, ^***^P < 0.001, paired *t* test. (**B**–**D**) IHC results related to significant genes. (**E**) Nonparametric test (*U* test) for continuous variables and risk groups in the training set; *P* < 0.05 represents significant difference. (**F**) *U* test for continuous variables and risk groups in the test set; *P* < 0.05 represents significant difference. (**G** and **H**) Chi-square test for discrete variables and risk groups. (**G**) Training set. (**H**) Test sets. *P* < 0.05 represents a significant difference.

**Figure 7:**
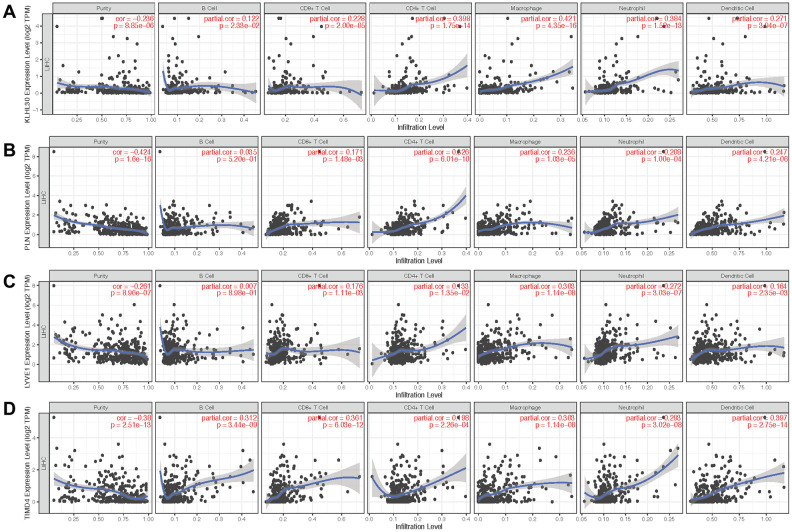
**Scatter plot of gene expression and immune cell content.** (**A**–**D**) show the relationship between the expression levels of KLHL30, PLN, LYVE1, and TIMD4 and the content of immune cells, respectively.

Next, qPCR experiments were used to check the change in gene expression level in normal cell line (THLE3) and LIHC cell line (SNU-423). As shown in Figure 6A, gene expression of KLHL30 and PLN significantly changed, while no significant difference was observed in the expression levels of LYVE1 and TIMD4 (Supplementary Figure 7). To some extent, the results of qPCR proved the difference in the expression of these genes in different cell lines. Moreover, the Human Protein Atlas database was used to further understand the functions of these significant genes in the ICRP score model. The related IHC results showed that the expression of PLN was upregulated in tumor tissues while the expression of LYVE1 and TIMD4 were down-regulated (Figure 6B–6D, KLHL30 was not found in this database), which indicated these genes do have expression variation in protein level during the development of liver cancer. Detailed information on IHC results was shown in Supplementary Table 2. Furthermore, the study explored whether the aforementioned significant factors had a strong correlation with the ICRP score. Hence, the chi-square test for discrete variables and *U* test for continuous variables were performed in both training and test sets. Age, Asian, bilirubin, and PLN had a strong difference between high–ICRP score and low–ICRP score groups, while grade was significant in the test set than in the training set (Figure 6E–6H). To observe the relationship of these four genes and immune infiltration, TIMER database (https://cistrome.shinyapps.io/timer/) was used to calculate the correlation between the genes and different immune cells to observe the relationship of these four genes with immune infiltration. As shown in Figure 7, KLHL30 expression was positively associated with the infiltration of B cells (Cor = 0.122, *P* = 0.023), CD8+ T cells (Cor = 0.228, *P* = 2e-05), CD4+ T cells (Cor = 0.398, *P* = 1.75e-14), macrophages (Cor = 0.421, *P* = 4.34e-16), neutrophils (Cor = 0.384, *P* = 1.52e-13), and dendritic cells (Cor = 0.271, *P* = 3.94e-07). The PLN expression was closely associated with the infiltration level of CD8+ T cells (Cor = 0.171, *P* = 0.001), CD4+ T cells (Cor = 0.326, *P* = 6.01e-10), macrophages (Cor = 0.236, *P* = 1.03e-05), neutrophils (Cor = 0.208, *P* = 0.0001), and dendritic cells (Cor = 0.247, *P* = 4.21e-06). The LYVE1 expression was positively related to the infiltration of CD8+ T cells (Cor = 0.176, *P* = 0.001), CD4+ T cells (Cor = 0.133, *P* = 0.013), macrophages (Cor = 0.303, *P* = 1.14e-08), neutrophils (Cor = 0.272, *P* = 3.03e-07), and dendritic cells (Cor = 0.164, *P* = 0.002). A positive correlation was found between the TIMD4 expression level and the infiltration of B cells (Cor = 0.312, *P* = 3.43e-09), CD8+ T cells (Cor = 0.361, *P* = 6.03e-12), CD4+ T cells (Cor = 0.198, *P* = 2.26e-4), macrophages (Cor = 0.303, *P* = 1.14e-08), neutrophils (Cor = 0.293, *P* = 3.02e-08), and dendritic cells (Cor = 0.397, *P* = 2.75e-14). The results indicated that the aforementioned four genes related to prognosis had a strong correlation with immune process, explaining why these genes could be used as biomarkers in LIHC prognosis. Previous studies [31] showed that the number of activated monocytes and plasma cells decreased and the numbers of B cells, CD4+ T cells, and CD8+ T cells increased in LIHC, compared with the healthy liver. This was consistent with the results of the present study. It is generally believed that B cells can be used as antigen-presenting cells to induce CD4+ T cell–dependent CD8+ memory T cells [32], thereby helping to control tumor invasion and metastasis.

## DISCUSSION

The prognostic model ICRP score built in this study provided a reference for the treatment of patients with LIHC. In this model, Asian, low age, and low grade were favorable factors for prognosis. High bilirubin was a disadvantage for prognosis, which may be because bilirubin reflected the degree of damage to liver cells during the occurrence of LIHC [33]. This was consistent with the results of ALBI [8] (ALBI score = log10 (0.66 × Bilirubin) − 0.085 × Albumin, where bilirubin is in μmol/L and albumin in g/L). The predictive power of the ICRP score was better than that of the AJCC stage and ALBI score mainly because of the introduction of four genes closely related to tumor stem cell content and immune cell content in LIHC.

Kelch-like protein 30 (KLHL30) is a 578-amino-acid protein containing 1 broad-complex, tramtrack, and bric-a-brac (BTB)/kelch-associated) domain, 1 BTB domain, and 6 kelch repeats [34]. The function of KLHL30 in mammals is unclear, but previous studies have shown that proteins containing BTB domains are often important in modifying the structure of chromosomes. Such proteins can usually control the dynamic changes in chromosomes and coordinate the completion of accurate mitosis [35]. Therefore, KLHL30 was involved in the division of cancer cells, and its high expression was a disadvantage for prognosis, as shown in the proposed model. Therefore, KLHL30 may be a potential prognostic marker.

The protein encoded by Phospholamban (PLN) gene is a major substrate for the cAMP-dependent protein kinase [36]. When the protein is phosphorylated, Ca^2+^- ATPase was activated, so that calcium ions in the cytoplasm were transported to the endoplasmic reticulum. A previous study showed [37] that the increase in the concentrations of basic calcium ions and transient calcium ions in the cytoplasm was involved in the cell cycle and cell proliferation. The high expression of PLN protein was obviously not conducive to this process, which was also the reason why the coefficient of PLN (−1.2207) in the regression model was negative. In addition, another study [38] found that the increase in the concentration of intracellular calcium ions had a special relationship with tumor metastasis. However, the specific mechanism still needs to be verified by further experiments.

Lymphatic vessel endothelial hyaluronan receptor 1 (LYVE1) encodes a type I integral membrane glycoprotein, which can bind to hyaluronan (hyaluronic acid, HA) on the plasma membrane to result in dendritic cell (DC) docking to lymphatic endothelial cells. DC docking is the key step prior to the endothelial transmigration into lymphatic vessels for immune activation. Consistent with the IHC results, some studies reported the low expression of LYVE1 in LIHC [39]. Meanwhile, LYVE1 marked lymphadenogenesis, which promoted tumor cell dissemination [40]. These reports supported the outcome of the present study that LYVE1 had a low expression in LIHC while its high expression might indicate poor prognosis.

T cell immunoglobulin and mucin domain containing 4 (TIMD4), also known as TIM4, is mainly expressed in antigen-presenting cells. Some recent studies reported that TIMD4 had a correlation with some malignant carcinomas and its upregulation might lead to poor prognosis, as in diffuse large B-cell lymphoma and non-small-cell carcinoma [41]. Li et al. [42] showed that the expression level of TIMD4 in glioma influenced the cancer tissues in different ways; low expression suppressed the growth and colony-forming ability of cancer cells, while high expression accelerated the growth and clonogenic potential of cancer cells. This report indicated that TIMD4 was involved in LIHC because its level in tumor tissues decreased when immune system worked, but a high expression of this gene might be associated with tumor recurrence and poor prognosis.

## CONCLUSIONS

LIHC data from TCGA, combined with the ESTIMATE algorithm and mRNAsi score, were used to find key genes closely related to the immune process and tumor stem cell content. An effective prognostic model ICRP score containing four genes and four clinical factors were established using these key genes and patient clinical factors. The verification of ROC curves and C-index implied that the proposed prognostic model was superior to the AJCC stage and ALBI score, indicating its huge application potential in clinic.

## MATERIALS AND METHODS

### Data collection and screening of differentially expressed genes

Liver cancer patient data from The TCGA (https://www.cancer.gov/tcga) database was used for analysis. RNA-seq data from 374 cancer tissue samples and 50 control tissue samples were obtained and then standardized by “Fragments Per Kilobase per Million” (FPKM). For genes with duplicate records, the average value of gene expression was calculated as the final value. In this process, the “limma” package in the R language was used. Genes whose average expression in all samples was less than 0.2 were excluded to make the screening of differentially expressed genes (DEGs) more reasonable. The Wilcoxon test was used to screen DEGs; genes with |log_2_foldchange| >1 and *P* <0.05 were identified as DEGs.

### Determination of tumor stem cell score

Malta et al [16] used one-class logistic regression machine learning algorithm to extract transcriptomic and epigenetic feature sets derived from nontransformed pluripotent stem cells and their differentiated progeny. They provided data on tumor stem cell scores from samples in the TCGA database. “mRNAsi” was the result based on all the expression profile data; “EREG-mRNAsi” was the result based on the expression of genes related to stem cell epigenetic regulation. These two scores ranged from 0 to 1, which was close to 1, indicating that the lower the degree of cell differentiation, the stronger the characteristics of stem cells. In this study, the samples were evaluated using mRNAsi scores, and subsequent WGCNA was performed.

### WGCNA of DEGs

Corresponding studies showed that gene regulatory networks obeyed scale-free networks. The WGCNA method [17] was proposed to meet this requirement. This method was used to find the significant gene modules in the specific genome profile. Different from the “hard cutoff,” WGCNA obtained the scale-free network by calculating the power value *β*, also known as “soft cutoff.” The Pearson correlation coefficient was calculated to construct gene co-expression matrix (1), and then this matrix was transformed into adjacent matrix using exponential adjacent equation (2). Topological overlap matrix was used to obtain the degree of association between genes (3) by setting gene module parameters: MaxBlocksize = 9000; deepSplit = 2; minModuleSize = 40; and mergeCutHeight = 0.40. Dissimilarity degree *d_ij_* (4) and dynamic fuzzy decision tree were first used to divide gene modules.

$$S_{ij}\;=\;cor(i,\;j)$$ (1)

$$\alpha_{ij}\;=\;|S_{ij}{|}^{\beta }$$ (2)

$$\omega \text{ij}=\frac{l_{ij}+\alpha_{ij}}{\min\;(k_{i},\;k_{j})+1-\alpha_{ij}}$$ (3)

$$d_{i}{}_{j}\;=\;1–\omega_{ij}$$ (4)

### DEGs related to immune processes

Yoshihara et al. [12] established the ESTIMATE method. This method used immune cell–related gene expression profiles, considered the screened DEGs as background genes, and performed gene-set enrichment analysis [18] (GSEA) on external samples to obtain “ImmuneScore” scores for external samples, which were used to evaluate the immune cell content of the samples. Adopting a similar process, the ESTIMATE algorithm was also used to calculate the “StromalScore” of a sample to assess the content of stromal cells in the sample. The ESTIMATE algorithm was performed to analyze 374 liver cancer samples from the TCGA database. These samples were grouped according to the median score of “ImmuneScore,” and DEGs were screened between high- and low-score groups. Log_2_|foldchange| >1 and *P* <0.05 were set as threshold values. For “StromalScore” scoring, the same process was repeated to screen out DEGs. The intersection genes of the DEGs of “ImmuneScore” and “StromalScore” were considered as genes closely related to the immune process of liver cancer.

### Gene ontology and kyoto encyclopedia of genes and genomes pathway enrichment analysis

GO (Gene Ontology) and KEGG (Kyoto Encyclopedia of Genes and Genomes) databases are open to public and provide the function annotations of gene sets, rendering a better understanding of their biological functions. GO enrichment analysis included three aspects [19], namely biological process, cellular component (CC), and molecular function (MF), and KEGG was mainly used to conduct pathway enrichment analysis [20]. These two methods were applied to analyze the DEGs screened, setting *P* value <0.05 as the cutoff. The R package “enrichplot,” “clusterProfiler,” and “ggplot” were used for all aforementioned processes.

### Establishment of the ICRP score

Of 374 liver cancer samples, samples with incomplete clinical information were removed, finally obtaining 225 samples containing complete clinical information. The gene expression data and clinical data of the samples were employed to perform prognostic analysis. A total of 225 samples were randomly divided into a training set (114 samples) and a test set (111 samples). The lasso regression [21] was carried out in the training set to eliminate the collinearity between factors, and the appropriate penalty coefficient was selected to minimize the partial likelihood deviance of the regression equation. The multivariate Cox proportional-hazards regression analysis [22, 23] was performed in the training set to build a prognostic model, named Immunity and Cancer-stem-cell Related Prognosis (ICRP) score, as follows:

$$ICRPscore\;=\;\sum_{l=1}^{N}Fi\times \beta i$$

where *Fi* is the value of the *i*th factor, *β* is the corresponding coefficient, and a high ICRP score represents a predicted poor prognosis.

### Verification of the ICRP score

The samples in the training and test sets were grouped according to the median value of the ICRP score in the training set, and then Kaplan–Meier [24] survival curves with log-rank test were plotted. The following other verifications on the training and test sets were performed at the same time to verify the validity of the ICRP score: (1) survival status of patients after 1, 3, and 5 years were predicted, and the receiver operating characteristic [25] (ROC) curves were plotted to calculate the AUC. (2) The C-index [26] was calculated to evaluate the predictive effect of the prognostic model. Furthermore, ROC curves based on the AJCC stage and the Albumin–Bilirubin (ALBI) score were also drawn for comparison with the existing prognostic methods. The C-index of the AJCC stage, the ALBI score, and the signature of chromosomal instability (CIN25) from specific genes (containing 25 genes)[27] were calculated and compared with the results of the proposed model.

In the present study, quantitative real-time polymerase chain reaction (qPCR) assay was used to verify the expression level of four genes. Total RNA was extracted from normal liver cells (THLE3) and cancer cells (SNU-423) using the TRIzol extraction method, and then the concentration was measured using a NanoDrop 2000 spectrophotometer (Thermo Scientific, USA). cDNA was produced using the RevertAid First Strand cDNA Synthesis Kit (Thermo Scientific), and the reverse transcription reaction was conducted using the ABI7900 system (Applied Biosystems, USA). Finally, the relative gene expression was calculated by the 2^-ΔΔCt^ method. The primer sequences used in the qPCR are shown in Supplementary Table 1. Each sample was measured in triplicate.

Simultaneously, the Human Protein Atlas database (http://www.proteinatlas.org) was used to observe the gene expression at the protein level for further understanding the function of key genes in the proposed prognostic model. The TIMER database (https://cistrome.shinyapps.io/timer/) was used to explore the relationship between gene expression and immune cell content.

The workflow of this study is shown in Figure 1A.

## Supplementary Material

Supplementary Figures

Supplementary Tables
