# Supplementary material for: Prognostic model of patients with liver cancer based on tumor stem cell content and immune process
Source: Aging (Albany NY). 2020 Aug 27;12(16):16555–78. doi: 10.18632/aging.103832 (PMC7485734; doi:10.18632/aging.103832)
Supplement: Supplementary Figures [file aging-12-103832-s002..pdf]

SUPPLEMENTARY FIGURES

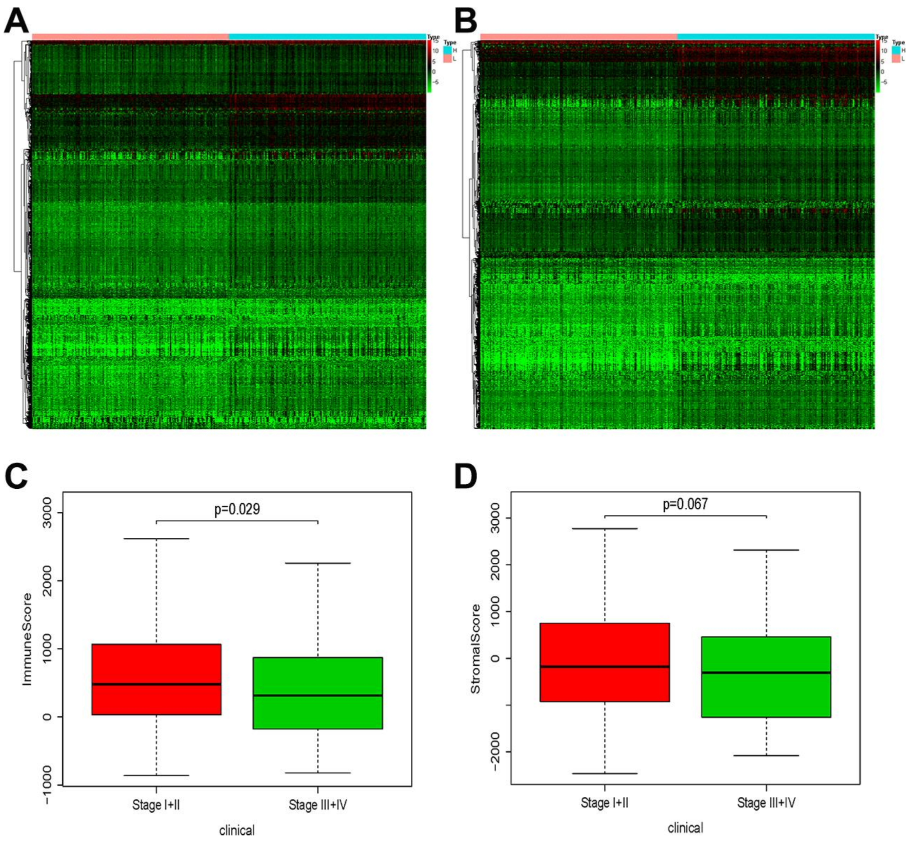

Supplementary Figure 1. Results of the ESTIMATE method. (**A**) Heatmap of DEGs for ImmuneScore. In the "Type," Cyan (H) denotes the high-score group and pink (L) denotes the low-score group. (**B**) Heatmap of DEGs for StromalScore. In the "Type," Cyan (H) denotes the high-score group and pink (L) denotes the low-score group. (**C**) The relationship between ImmuneScore and tumor stage was tested using the Wilcoxon test. (**D**) The relationship between StromalScore and tumor stage was tested using the Wilcoxon test.

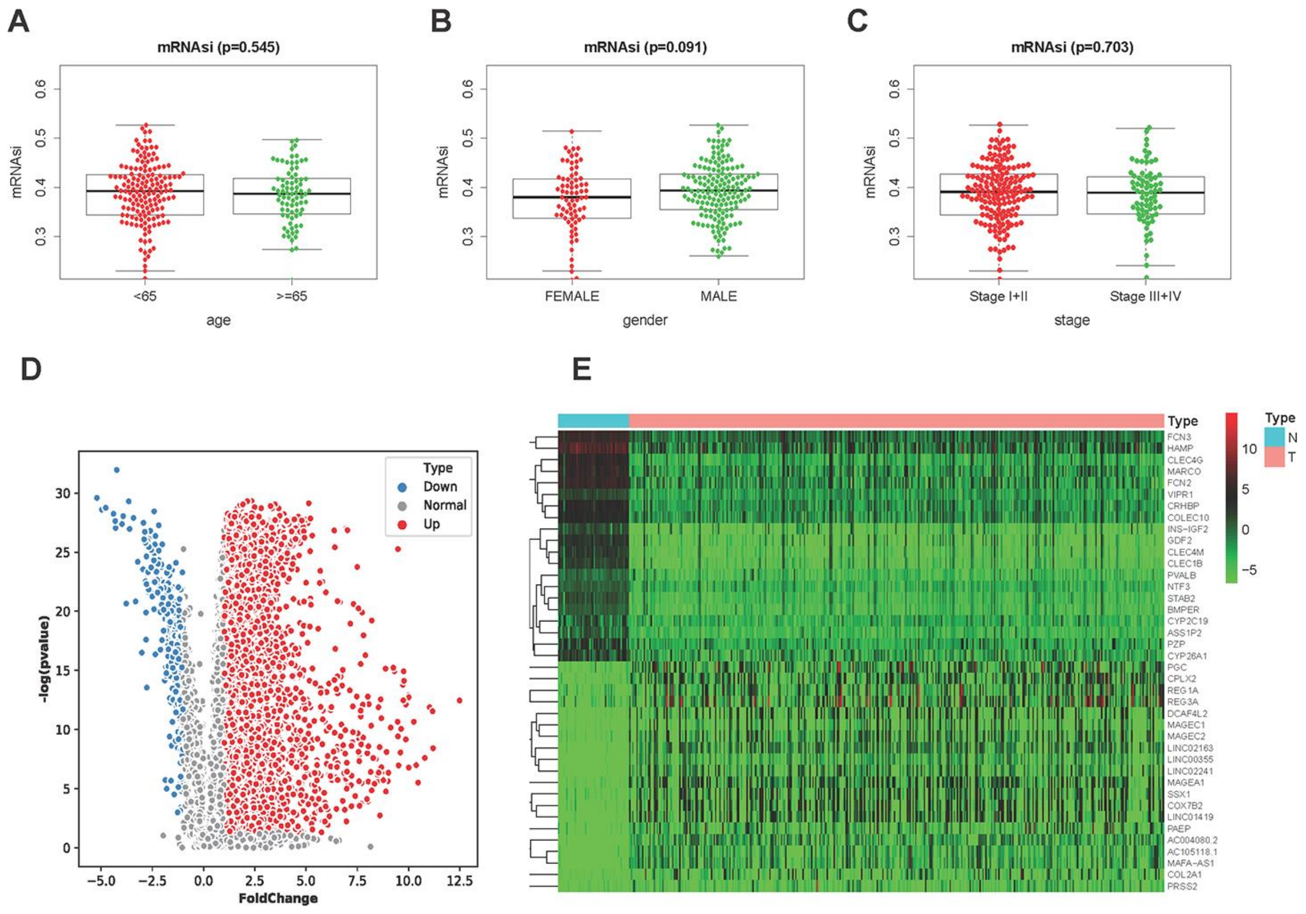

Supplementary Figure 2. The relationship between mRNAsi and clinical traits and DEGs in patients with LIHC and normal samples. (**A**) The relationship between mRNAsi and age. The Wilcoxon test was used. (**B**) Relationship between mRNAsi and sex. The Wilcoxon test was used. (**C**) Relationship between mRNAsi and stage. The Wilcoxon test was used. (**D**) Volcano figure of DEGs from patients with LIHC and normal samples. (**E**) Heat map of DEGs. The figure shows the expression of the top 40 genes in |logFC|. In the "Type," cyan denotes normal samples and pink denotes the tumor samples.

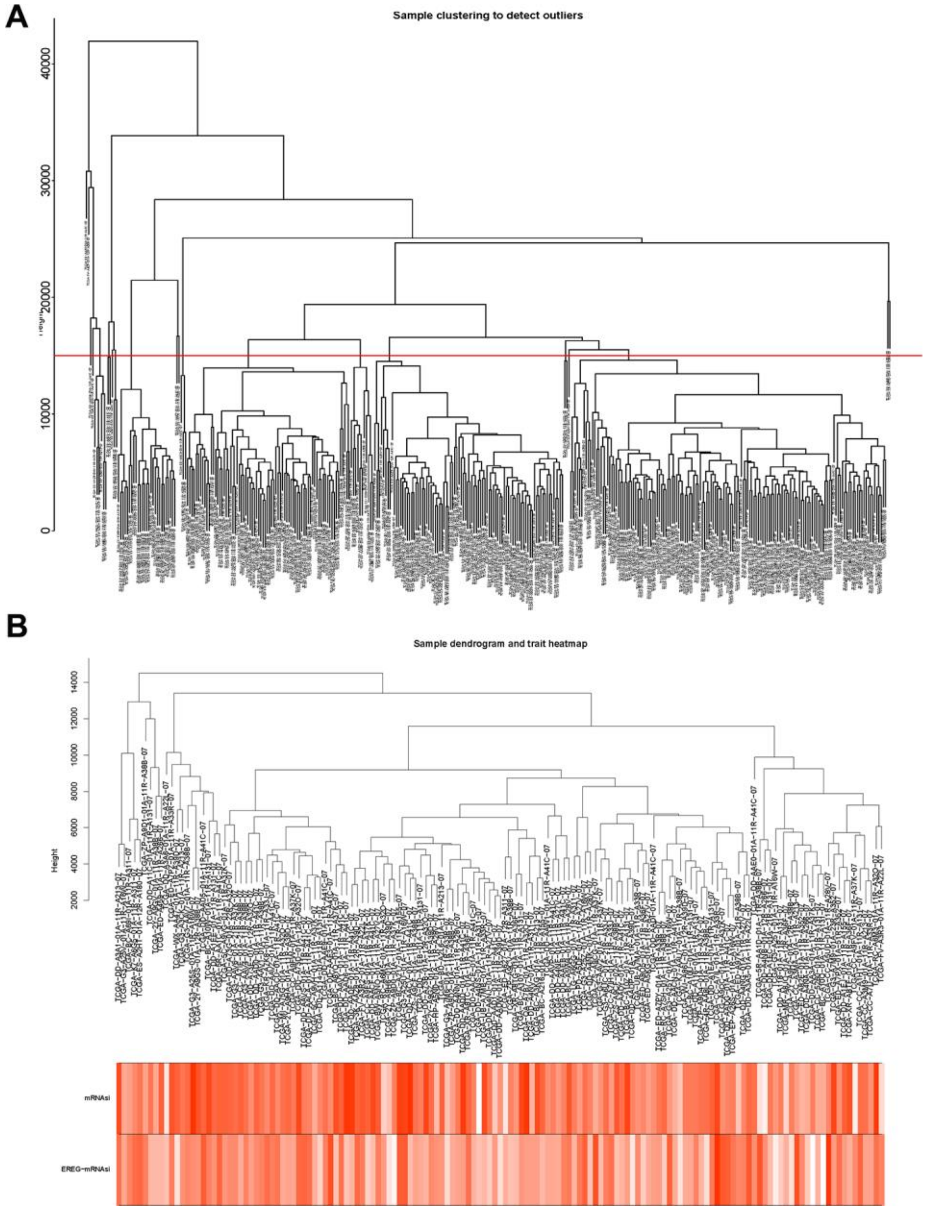

Supplementary Figure 3. Pretreatment of samples before WGCNA. (**A**) The result of hierarchical clustering before excluding samples. Criteria for excluding samples: the red line is 15,000. (**B**) Results of hierarchical clustering after excluding samples. The heatmap shows the mRNAsi score and EREG-mRNAsi score.

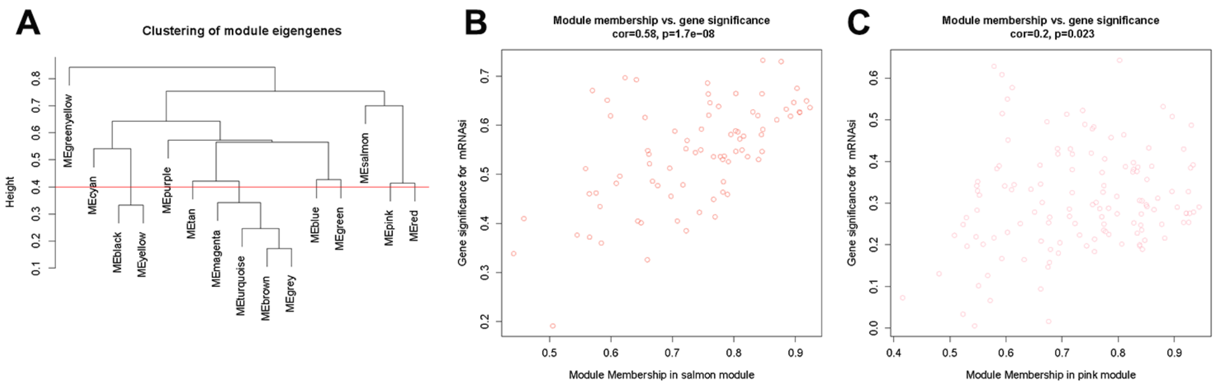

Supplementary Figure 4. Results of WGCNA analysis. (**A**) Gene module clustering results. Modules with height less than 0.4 (red line) were merged. (**B**) Scatter plot of gene importance in the salmon module. The *x*-axis is the Pearson correlation coefficient of gene expression and the first principal component of the salmon module. The *y*-axis is the correlation coefficient of gene expression and mRNAsi of the sample. Genes in the upper right are generally thought to be involved in this module. (**C**) Scatter plot of gene importance in the pink module.

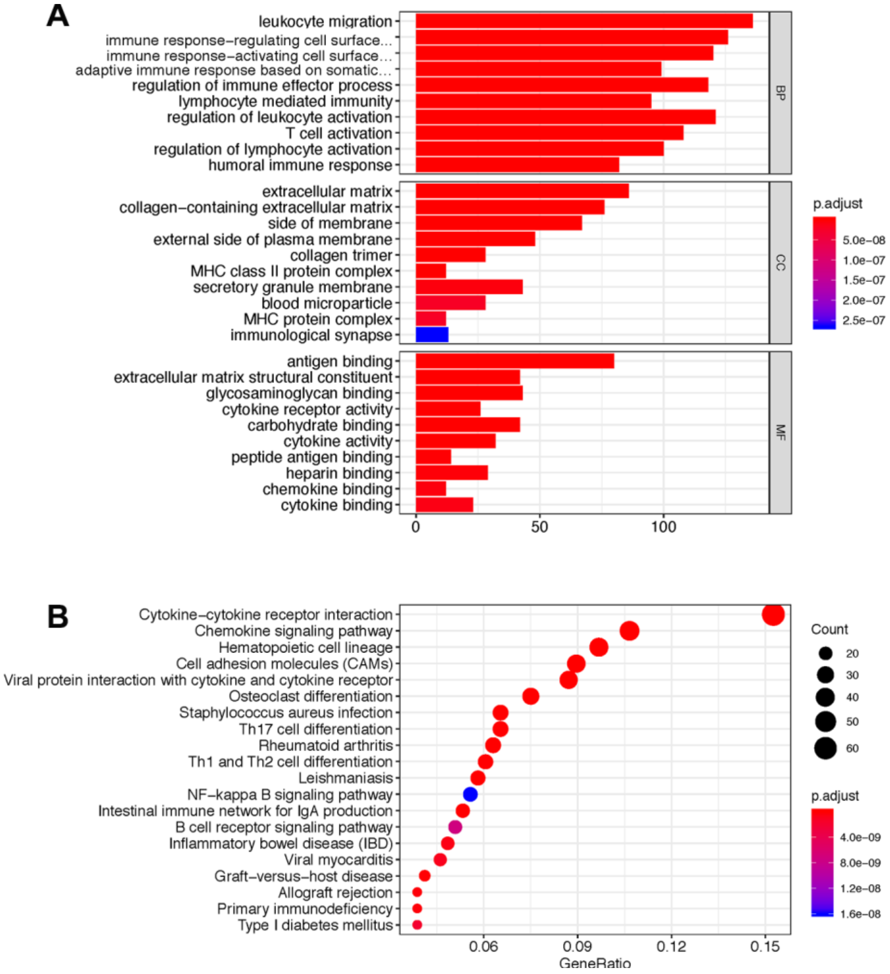

Supplementary Figure 5. GO and KEGG analyses of DEGs from ESTIMATE algorithm. (**A**) GO analysis; (**B**) KEGG analysis.

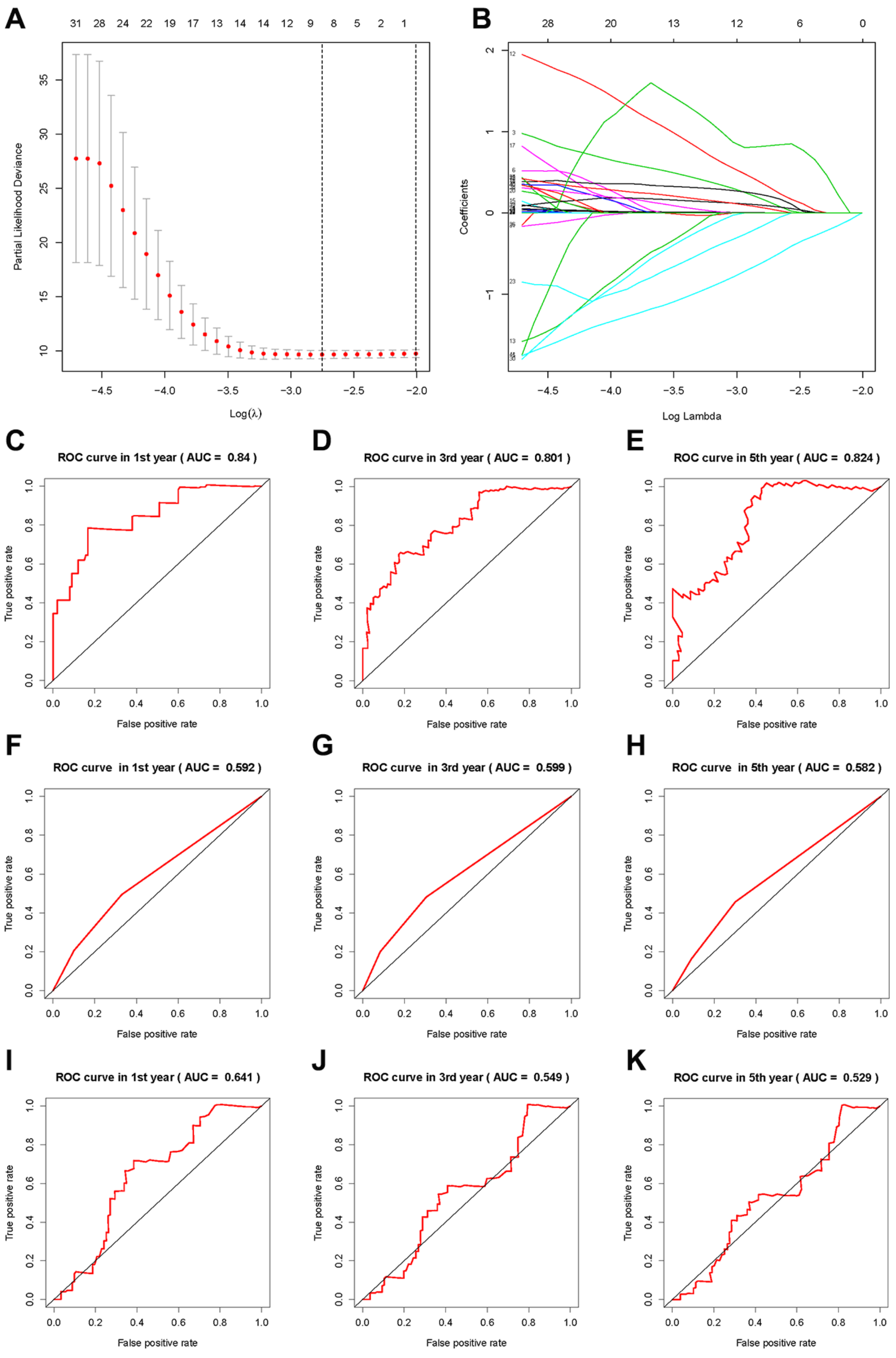

Supplementary Figure 6. Results of lasso regression and ROC curves during prognosis. (**A**) The result of cross-validation. The *y*-axis is partial likelihood deviance. The *x*-axis (bottom) is log (λ), and the *x*-axis (up) is the number of remaining variables. The dashed line gives the λ value when the partial likelihood deviance is minimum. (**B**) The *y*-axis is the coefficient of the variables in the lasso regression equation. (**C**–**E**) ROC curve of the ICRP score forecast result after 1, 3, and 5 years in the training set. (**F**–**H**) ROC curve of AJCC stage forecast result after 1, 3, and 5 years in the training set. (**I**–**K**) ROC curve of ALBI score prediction results after 1, 3, and 5 years in the training set.

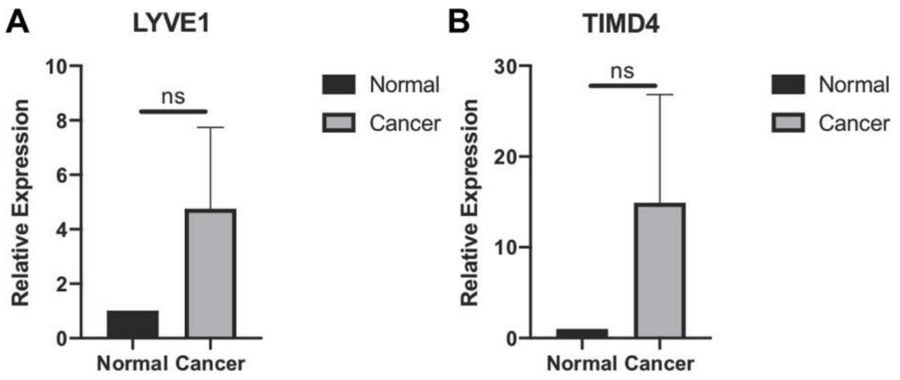

Supplementary Figure 7. (**A**, **B**) QPCR analysis of LYVE1 and TIMD4 in the normal liver cell line and LIHC cell line (n = 3). No significance, paired-sample t test.
