# Supplementary material for: Prognostic model of patients with liver cancer based on tumor stem cell content and immune process
Source: Aging (Albany NY). 2020 Aug 27;12(16):16555–78. doi: 10.18632/aging.103832 (PMC7485734; doi:10.18632/aging.103832)
Supplement: Supplementary Tables [file aging-12-103832-s001..pdf]

SUPPLEMENTARY TABLES

Supplementary Table 1. Sequence of primers used for real-time quantitative PCR.

KLHL30	Forward primer	5‘-AGCTATGACCCCTACACGGA-3‘
	Reverse primer	5‘-CGATCACACTCCACGCATCT-3‘
LYVE1	Forward primer	5‘-AGCTTTGAAACTTGCAGCTATGG-3‘
	Reverse primer	5‘-TCCAAATCAGGACACCCACC-3‘
PLN	Forward primer	5‘-ATCACAGCTGCCAAGGCTA-3‘
	Reverse primer	5‘-AGCTGAGCGAGTGAGGTATTG-3‘
TIMD4	Forward primer	5‘-AGCAAACACGTGCCTTTCAC-3‘
	Reverse primer	5‘-GGGTATTCCATCCATCTGTCCT-3‘
ACTB	Forward primer	5‘-CATGTACGTTGCTATCCAGGC-3‘
	Reverse primer	5‘-CTCCTTAATGTCACGCACGAT-3‘

Supplementary Table 2. Detailed information of IHC results.

Gene	Type	Patient ID	Gender	Age	Staining	Intensity	Quantity
PLN	Normal	2429	Male	55	Not detected	Negative	None
	Tumor	3625	Male	59	Low	Weak	>75%
LYVE1	Normal	3402	Female	54	Low	Moderate	<25%
	Tumor	983	Female	53	Not detected	Negative	None
TIMD4	Normal	3402	Female	54	Low	Weak	>75%
	Tumor	2399	Female	52	Not detected	Negative	None
